# Downregulation of lncRNA Miat contributes to the protective effect of electroacupuncture against myocardial fibrosis

**DOI:** 10.1186/s13020-022-00615-6

**Published:** 2022-05-17

**Authors:** Wenchuan Qi, Xiang Li, Yanrong Ren, Xueying Liu, Hongjuan Fu, Xiao Wang, Xiao Li, Jian Xiong, Qianhua Zheng, Dingjun Cai, Fanrong Liang

**Affiliations:** 1 College of Acupuncture, Moxibustion and Tuina, Chengdu University of Traditional Chinese Medicine, Chengdu, 610075 Sichuan China; 2 College of Acupuncture, Moxibustion and Tuina, Shanxi University of Traditional Chinese Medicine, Jinzhong, 030002 Shanxi China

**Keywords:** Myocardial fibrosis, Electroacupuncture, lncRNA Miat, CTGF, TGF-β1, Heterodimer PPARG–RXRA

## Abstract

**Background:**

Myocardial fibrosis changes the structure of myocardium, leads to cardiac dysfunction and induces arrhythmia and cardiac ischemia, threatening patients’ lives. Electroacupuncture at PC6 (Neiguan) was previously found to inhibit myocardial fibrosis. Long non-coding RNAs (lncRNAs) play a variety of regulatory functions in myocardial fibrosis, but whether electroacupuncture can inhibit myocardial fibrosis by regulating lncRNA has rarely been reported.

**Methods:**

In this study, we constructed myocardial fibrosis rat models using isoproterenol (ISO) and treated rats with electroacupuncture at PC6 point and non-point as control. Hematoxylin–eosin, Masson and Sirius Red staining were performed to assess the pathological changes and collagen deposition. The expression of fibrosis-related markers in rat myocardial tissue were detected by RT-qPCR and Western blot. Miat, an important long non-coding RNA, was selected to study the regulation of myocardial fibrosis by electroacupuncture at the transcriptional and post-transcriptional levels. In post-transcriptional level, we explored the myocardial fibrosis regulation effect of Miat on the sponge effect of miR-133a-3p. At the transcriptional level, we studied the formation of heterodimer PPARG–RXRA complex and promotion of the TGF-β1 transcription.

**Results:**

Miat was overexpressed by ISO injection in rats. We found that Miat can play a dual regulatory role in myocardial fibrosis. Miat can sponge miR-133a-3p in an Ago2-dependent manner, reduce the binding of miR-133a-3p target to the 3ʹUTR region of CTGF mRNA and improve the protein expression level of CTGF. In addition, it can also directly bind with PPARG protein, inhibit the formation of heterodimer PPARG–RXRA complex and then promote the transcription of TGF-β1. Electroacupuncture at PC6 point, but not at non-points, can reduce the expression of Miat, thus inhibiting the expression of CTGF and TGF-β1 and inhibiting myocardial fibrosis.

**Conclusion:**

We revealed that electroacupuncture at PC6 point can inhibit the process of myocardial fibrosis by reducing the expression of lncRNA Miat, which is a potential therapeutic method for myocardial fibrosis.

**Supplementary Information:**

The online version contains supplementary material available at 10.1186/s13020-022-00615-6.

## Introduction

Myocardial fibrosis refers to the excessive accumulation of collagen fibers in the tissue structure of the myocardium, while its collagen concentration or collagen volume fraction is significantly increases [1, 2]. In recent years, many studies have shown that myocardial fibrosis can occur in many cardiovascular diseases, such as hypertension [3], myocardial infarction [4] and heart failure [5, 6], and can even cause sudden death [7]. Myocardial fibrosis involves a variety of original causes, and its regulatory mechanism is affected by many factors [8–10]. Generally speaking, myocardial fibrosis is the result of an imbalance in collagen synthesis and degradation [11–13]. Transforming growth factor-β1 (TGF-β1) is a multifunctional protein peptide, which can increase the synthesis of collagen-based interstitial proteins [14, 15]. TGF-β1 can increase the expression of type I and type II collagen mRNA in cultured cells in vitro and increase the stability of type I collagen mRNA [16, 17]. Recent studies have also found that there is a close interaction between TGF-β1 and Ang II [18, 19]. In the course of myocardial fibrosis, Ang II increases the expression of the TGF-β1 gene [20, 21], while TGF-β1 inhibits the degradation of extracellular matrix and increases the expression of extracellular matrix mRNA and protein synthesis [22, 23]. Connective tissue growth factor (CTGF) is a secreted peptide, which is rich in cysteine, widely present in human tissues and organs, and is related to diseases such as atherosclerosis and organ fibrosis [24]. CTGF share many biological functions with TGF-β1, and is the downstream product of TGF-β1 [25, 26]. Its abnormal expression plays an important role in the myocardial fibrosis process [27, 28]. Blocking the expression of TGF-β1 and CTGF or weakening their activity may be an effective means to prevent further development of myocardial fibrosis.

Long non-coding RNA (lncRNA) is defined as an RNA that is more than 200 nucleotides in length but lacks protein coding potential [29–31]. LncRNA regulates gene expression at multiple levels, including epigenetics, transcriptional regulation and post-transcriptional regulation [32]. LncRNA plays a key role in the occurrence and development of myocardial fibrosis [33, 34]. However, the mechanisms of lncRNA action in myocardial fibrosis have received relatively limited attention. Recent studies have shown that the lncRNA myocardial infarction associated transcript (Miat) is involved in the pathological process of many diseases, including diabetic retinopathy [35], myocardial infarction [36], microvascular dysfunction [37] and cardiac hypertrophy [38]. LncRNA cardiac hypertrophy-related factor (CHRF) is up-regulated in heart hypertrophy, and CHRF regulates cardiac hypertrophy by targeting miR-489 [39]. Valsartan et al. found that by down-regulating CHRF, it can inhibit the TGF-β signaling pathway and improve doxorubicin-induced heart failure [40].

Recently, researchers have studied the effect of lncRNA PFL on myocardial fibrosis. PFL promotes the cell proliferation and fibrosis of cardiac fibroblasts by acting as a competitive endogenous RNA of let-7d [41]. The lncRNA RASSF1-AS1 encoded by the antisense strand of the *RASSF1A* gene was significantly overexpressed in the isoproterenol (ISO)-induced mice model. RASSF1-AS1 binds to RASSF1 mRNA to promote NF-κB activation and inhibit the translation of RASSF1A, thus exacerbating myocardial fibrosis in mice, indicating a potential application of RASSF1-AS1 as a therapy target for myocardial fibrosis [42]. The expression of myocardial specific lncRNA Colorectal Neoplasia Differentially Expressed (CRNDE) is negatively correlated with the expression of genes related to myocardial fibrosis. CRNDE suppresses myocardial fibrosis by inhibiting Smad3 [43]. LncRNAs have various effects on myocardial fibrosis, which need to be further studied, the therapeutic methods of regulating lncRNA also need to be further explored.

Currently, there is no therapeutic drug that can effectively control the fibrotic reaction, which is accompanied by many side effects. Therefore, the discovery of alternative therapeutic methods for myocardial fibrosis is of great significance in clinical practice. Electroacupuncture of PC6, either preconditioning or after ischemia–reperfusion injury, reduced mortality in mice [44] and rats [45, 46]. In addition, pretreatment with electroacupuncture at PC6 reduced the size of myocardial infarction caused by coronary artery ligation in rats [47]. Electroacupuncture at PC6 after coronary artery ligation reduced cardiac scar area and improved angiogenesis in rats [48]. Myocardial fibrosis causes abnormalities in the cardiac function, metabolism and conduction, which can lead to heart failure and various arrhythmias [49]. Electroacupuncture at PC6 inhibited myocardial fibrosis on hypertension-induced myocardial fibrosis in spontaneously hypertensive rats (SHRs), which may be mediated by down-regulation of the enhanced Ang II-TGF-β1-CTGF/TNF-α pathway and up-regulation of the reduced MMP-9 expression [50].

In this study, we used ISO to construct rat models with myocardial fibrosis for experimental study, and studied the anti-fibrosis effect of electroacupuncture and its potential mechanism in regulating lncRNA Miat.

## Materials and methods

### Animals and ethics

Male Sprague-Dawley (SD) rats weighing 250 ± 30 g (SCXK (Chuan) 2015-030; Dashuo Co., Ltd., Chengdu, China) were housed in a controlled condition (12:12 h light: dark cycle, 25 ± 2 °C, 50% ± 5% relative humidity) with food and water ad libitum. All experimental procedures were approved by the Chengdu University of Traditional Chinese Medicine Animal Welfare and Ethics Committee and conformed to the standards of the International Council for Laboratory Animal Science.

### Animal model establishment

SD rats were injected with isoproterenol (ISO) to cause myocardial fibrosis due to chronic myocardial ischemia. Male SD rats were randomized into four groups (n = 10): normal saline (NS) control, isoproterenol injection (ISO) group, ISO + electroacupuncture (EA) group and ISO + non-acupoint (NA) control. Isoproterenol (2 mg/kg;) was injected intraperitoneally (i.p.) for 2 weeks, to establish the myocardial fibrosis rat model. Control mice were injected with normal saline at equivalent volume. At the end of each experiment, the rats were sacrificed with high dose of isoflurane.

### Electroacupuncture treatment

Prior to electroacupuncture treatment, all rats were restrained using the same method. The animals in both ISO + EA (n = 10) and ISO + NA (n = 10) groups received electroacupuncture treatment for 20 min daily for a total of 2 weeks. Two acupuncture needles (15 mm long and 0.3 mm in diameter) were inserted at a depth of 2–3 mm into the PC6 acupoints with an electrical stimulator (Han’s acupoint nerve stimulator, HANS-200, Nanjing, China) at a frequency of 2/100 Hz and an intensity level of 1 mA for 20 min. Researches on the specificity of acupoints need a contrast of non-acupoint. Select a point at the rat tail as the non-meridian and non-acupoint control point, which can better avoid the meridians and acupoints [46]. Therefore, the same treatments were applied to the base of the tail in the NA group rats. After the treatment, we randomly selected six rats (n = 6) in each group for subsequent experiments.

### Histopathological analysis

The heart was fixed in neutral paraformaldehyde solution (4%) (Solarbio, Beijing, China) for tissue fixation. The organs were cut into appropriate sections, washed, dehydrated and embedded to make tissue wax. The samples were then sectioned (4-µm thickness) and separately stained with hematoxylin and eosin (H&E) (Solarbio), Masson trichrome (Solarbio) and Sirius-Red staining (Solarbio), according to the manufacturer’s protocols. To analyze the extent of damage, images were captured using a microscope (Nikon, Japan). The area of damage in the heart tissues was determined using Image J (version 1.8.0) software.

### Western blot

For the Western blot analysis, the protein was extracted from the rats’ myocardial tissue using a Tissue Protein Extraction Kit (Beyotime Biotechnology, Shanghai, China) according to the protocol provided by the manufacturer. RIPA lysis buffer (Servicebio, Wuhan, China) was used to extract protein from H9c2 cells. Protein concentrations were quantified using the Bicinchoninic Acid (BCA) Protein Assay Kit (Thermo Fisher Scientific, MA, USA). Total proteins obtained from cardiac tissues were loaded onto 10% sulfate-polyacrylamide gel electrophoresis and transferred to polyvinylidene fluoride membranes. After blocking with 5% non-fat milk for 1 h at room temperature, the membranes were incubated at 4 °C overnight with Collagen I, Collagen III, CTGF, TGF-β1, PPARG, RXRA and Actin antibody, which were purchased from ProteinTech (Chicago, IL, USA), and ACTIN was used as the loading control. After washing with Tris-buffered saline/0.1% Tween 20 (TBST) for 5 times, the membranes were incubated with the secondary antibodies for 1 h at room temperature. Goat anti-mouse IgG (Catalogue no. SA00001-1) and goat anti-rabbit IgG (Catalogue no. SA00001-2) antibodies were purchased from ProteinTech (Chicago, IL, USA). Mouse anti-rabbit IgG LCS (Catalogue no. A25022) antibody was purchased from Abbkine (Wuhan, China). Immunoreactive bands were visualized using Immobilon ECL Ultra Western HRP Substrate (Merck, USA). The intensity of the bands was assessed using the Image Lab software from Bio-Rad (Bio-Rad, Hercules, CA, USA).

### H9c2 and HEK293 cells culture

H9c2 rat embryonic heart myoblast cells and HEK293 human embryonic kidney epithelial cells were purchased from Procell Life Science Technology Co. Ltd. (Wuhan, China) and cultured in high glucose Dulbecco’s Modified Eagle Medium (DMEM) (Hyclone, Logan, UT, USA) with 10% fetal bovine serum (FBS; Gibco, USA) and 1% of penicillin–streptomycin (Hyclone). All the cells were incubated in a humidified incubator at 37 °C under an atmosphere of 95% air and 5% CO_2_.

### Isolation of cytoplasmic and nuclear RNA

Nuclear and cytoplasmic components were separated using the Nuclear and Cytoplasmic Protein Extraction Kit (Beyotime, Shanghai, China) according to the manufacturer’s instructions. RiboLock RNase Inhibitor 100 U/mL (Thermo) was added to the lysis buffer from the kit before the experiment. The nuclear precipitate was directly resuspended in TRIzol reagent and subjected to RNA extraction.

### Plasmids and miRNAs transfection

H9c2 or HEK293 cells were plated in 6-well culture plates at a density of 2 × 10^5^ cells per well for 24 h. Transfections were performed using Lipofectamine^®^ 2000 (Invitrogen, Thermo Fisher Scientific, Inc) according to the manufacturer’s instructions. Plasmid DNA or siRNAs were diluted in Opti-MEM (Gibco, Thermo Fisher Scientific, Inc.) and mixed with pre-prepared solutions of Lipofectamine^®^ 2000 for 5 min at 25 °C. Thereafter, 250 μL of the complex solutions were added to each well, and the cells were slightly agitated to mix. Cells were then incubated for 4–6 h in a humidified incubator (5% CO_2_, 37 °C) before adding fresh media. Next, transfections were repeated at 24 h intervals (48 h and 72 h, as indicated). The Miat expression plasmid and siRNAs target for lncRNA Miat were designed and purchased from TSINGKE Biological Technology Co., Ltd. Control rno-miRNA (rno-miRNA-NC), rno-mir-133a-3p mimic and rno-mir-133a-3p inhibitors were purchased from RiboBio (Guangzhou, China).

### Quantitative reverse transcription-PCR (RT-qPCR)

Total RNA samples were extracted from cultured H9c2 cells or the left ventricular region of the heart using Trizol reagent (Invitrogen, USA). For each sample, 0.5 μg of total RNA was converted to cDNA using a cDNA reverse transcription kit (Thermo). The expression levels of mRNA and miRNA were analyzed by RT-qPCR reactions using the SYBR Green I RT-qPCR Easy™ (Foregene, Chengdu, China) and a BIOER Detection System (Bioer Technology Co. Ltd. Hangzhou, China). The RNA levels of Miat, miR-133a, collagen I (Col I) and collagen III (Col III) were detected by the SYBR Green method. After cyclic responses, cyclic thresholds (Ct) were determined, and the relative quantitative expression levels of Miat, miR-133a and mRNA were calculated using the method of 2^−ΔΔCq^ and normalized to ACTIN or U6 as internal controls. The primer sequences were synthesized by Tsingke, Chengdu. The primers of miR-133a-3p and U6 were purchased from RiboBio (Guangzhou, China). The sequences of the RT-qPCR Primers and siRNA oligonucleotides are listed in Additional file 1: Table S1.

### Dual luciferase reporter gene assay

Bioinformatics analysis predicted, as reported in literature, that lncRNA Miat contained a normative binding site for miR-133a-3p. Meanwhile, the analysis also showed that CTGF was a potential target gene of miR-133A-3p. To verify whether they can interact directly, we constructed the recombinant plasmid pmirGLO-CTGF-3ʹUTR (WT) and pmirGLO-CTGF-3ʹUTR (MUT). HEK293 cells were seeded into 24-well plates and co-transfected with luciferase reporter plasmids and miR-133a-3p mimics or miR-NC using Lipofectamine^®^2000 (Invitrogen) according to the manufacturer’s instructions. At 48 h after transfection, the luciferase activity of each group was detected using the dual luciferase reporter gene assay kit (Keygen, Nanjing, China). To test the TGF-β1 promoter activity, the promoter region of TGF-β1 was inserted into the pGL3 luciferase reporter vector (Progema, Madison, WI, USA). Recombinant Plasmid pGL3-TGF-β1 was extracted using EndoFree Mini Plasmid Kit II (Tiangen, Beijing). To verify the involvement of Miat in regulating TGF-β1 promoter activity, the plasmid pGL3-TGF-β1 (800 ng) was co-transfected with the plasmid PRL-TK (20 ng) using Lipofectamine^®^ 2000 according to the manufacturer’s protocol. At the same time, lncRNA Miat was overexpressed or knockdown as previously described. At 48 h after transfection, luciferase activity was measured using the dual-luciferase reporter gene assay system (Keygen) 3 times in each group.

### RNA immunoprecipitation (RIP) and co-immunoprecipitation (co-IP) assays

H9c2 cell lysates from 1 × 10^6^ cells were combined with 1 mL IP buffer (25 mM Tris-Cl [pH 7.4], 150 mM NaCl, 0.5% NP-40, 0.5 mM DTT and 1 × complete protease inhibitors [Roche]) supplemented with 100 U/mL RNase Inhibitor (Thermo) and subjected to immunoprecipitation with 2 μg PPARG antibody or IgG at 4 °C overnight with rotation. The immunoprecipitates were digested with proteinase K (Thermo Scientific), and then the immunoprecipitated RNA was recovered using TRIzol reagent (Thermo Scientific). cDNA was synthesized with primers using the RevertAid RT Kit (Thermo Scientific) and subjected to RT-qPCR for Miat transcripts and miR-133a-3p. For co-immunoprecipitation assays, cell lysates were incubated with PPARG antibody 2 μg overnight at 4 °C, and then with protein A/G plus agarose beads (20 μL/sample) for 1 h at 4 °C. Immunoprecipitants were collected by centrifugation and washed 4 times with lysis buffer, and then the beads were precipitated through centrifugation and re-suspended in 2× SDS loading buffer. The protein present in the co-IP material was examined by Western blot.

### Statistical analysis

All data are expressed as the mean ± SD. The significance of differences between two groups was determined using the Student’s unpaired t test. Comparisons between groups were performed using one-way ANOVA (Tukey’s multiple comparisons test). Differences with P < 0.05 were considered to be statistically significant.

## Results

### Electroacupuncture at PC6 reduces myocardial fibrosis

**Fig. 1:**
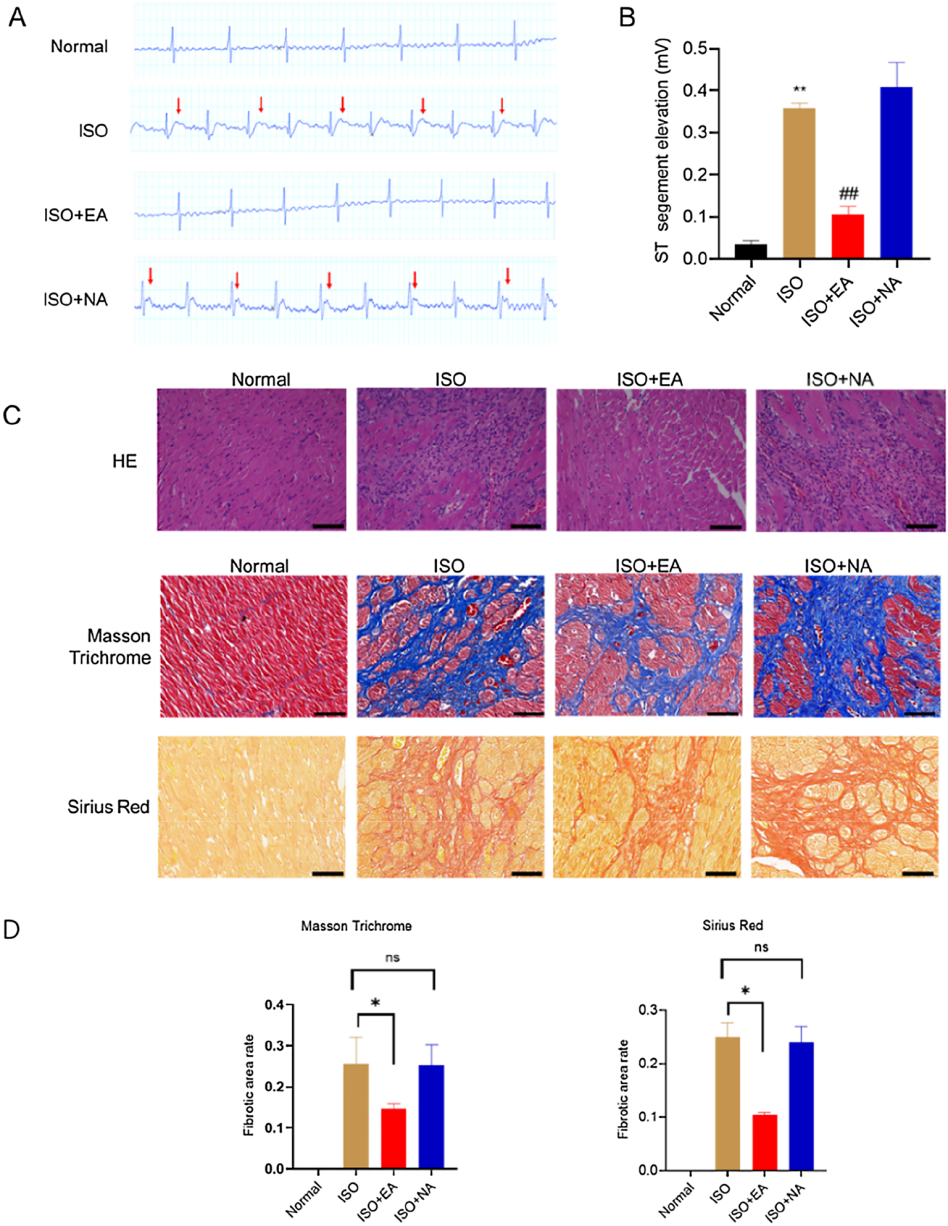
Effects of the electroacupuncture treatment on ISO-induced myocardial fibrosis. **A** Representative electrogastrogram (EGG) recordings and **B** Changes in rat ECG-ST (mV, **A**) of EGG in NS, ISO, ISO + EA and ISO + NA groups (n = 6 in each group). **C** Samples of myocardial tissue stained with hematoxylin and eosin (H&E) (bar = 100 μm), Masson’s trichrome (Tricon), and Sirius red. **D** Percent area fibrosis (Mason staining) and (Sirius staining) in the indicated groups (bar = 50 μm) (n = 6 per group)

Injecting ISO can cause myocardial ischemia, resulting in myocardial ischemia injury and myocardial fibrosis [51]. The representative ECG diagrams were selected for the analysis of myocardial ischemia injury. Electroacupuncture at PC6 (ISO + EA) significantly reversed the S-T segment change induced by ISO infusion. Conversely, electroacupuncture of the tail (ISO + NA group) demonstrated no such potential to decrease the S-T segment change (Fig. 1A and B). After 2 weeks of treatment, the hearts were harvested to quantify myocardial fibrosis. We first used HE staining to evaluate the myocardial tissue. Compared with the NA group, myocardial fibers in the ISO model group were deformed and disordered with striated muscle disappeared, cytoplasm was vacuolated to varying degrees, and inflammatory cell infiltration was observed in some myocardial tissues. In addition, the pathological damage of myocardial tissue was partially weakened in the electroacupuncture group, but not significantly weakened in the non-acupoint (NA) group. Masson collagen specific staining (normal myocardium is red, fibrosis area is blue) showed that compared with normal control group, collagen accumulation and the fibrosis area in the model group were significantly increased. In addition, the staining results of Sirius red were similar to those of Masson. Based on these results, we found that electroacupuncture PC6 attenuated myocardial fibrosis.

### Involvement of Collagen I/III, CTGF, TGF-b1 in the electroacupuncture-induced antifibrosis effects

**Fig. 2:**
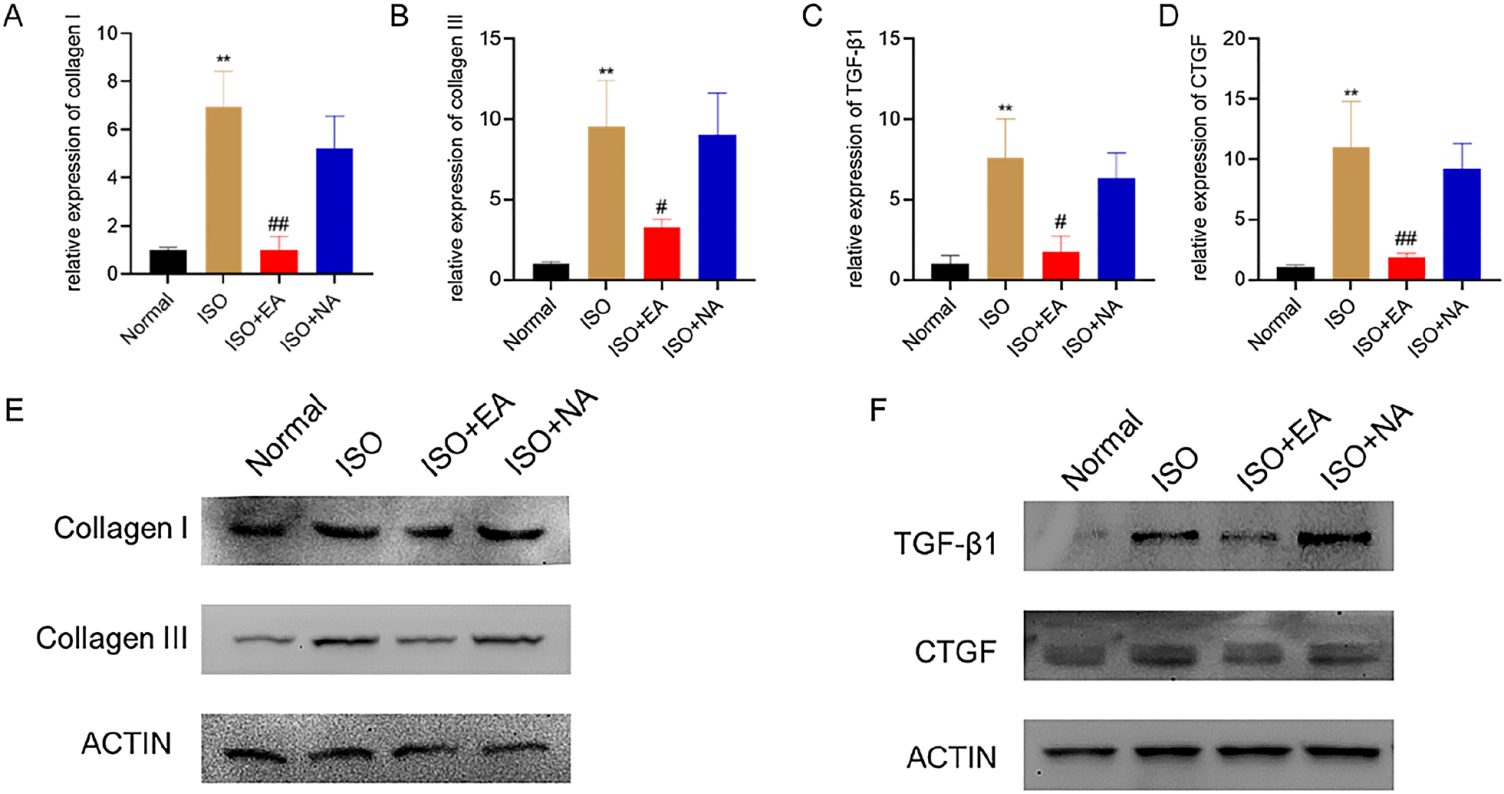
Effects of electroacupuncture on the level of myocardial fibrosis markers. **A**–**D** RT-qPCR was performed to determine the mRNA expression of Collagen I, Collagen III, TGF-β1 and CTGF in rat heart tissues (n = 6 rats in each group). **E**, **F** Western blot was performed to determine the protein expression of Collagen I, Collagen III, TGF-β1 and CTGF in rat heart tissues, ACTIN was used as loading control. Results are presented as mean ± SD. *P < 0.05, **P < 0.01

The expression of markers related to fibrosis in rat myocardial tissue was detected by RT-qPCR and Western blot. The mRNA expression levels of fibrosis related genes type I collagen, type III collagen, CTGF and TGF-β1 were significantly increased in the ISO rats’ myocardial tissue (Fig. 2A–D). The expression levels of these mRNAs were decreased by electroacupuncture at PC6. Similar results were observed in the expression of fibrosis-related protein, but electroacupuncture in NA had no significant effect (Fig. 2E and F). These results suggest that electroacupuncture at PC6 can inhibit myocardial fibrosis in ISO rats by inhibiting the expression of type I collagen, type III collagen, TGF-β1 and CTGF.

### Electroacupuncture can regulate the expression of lncRNA Miat

**Fig. 3:**
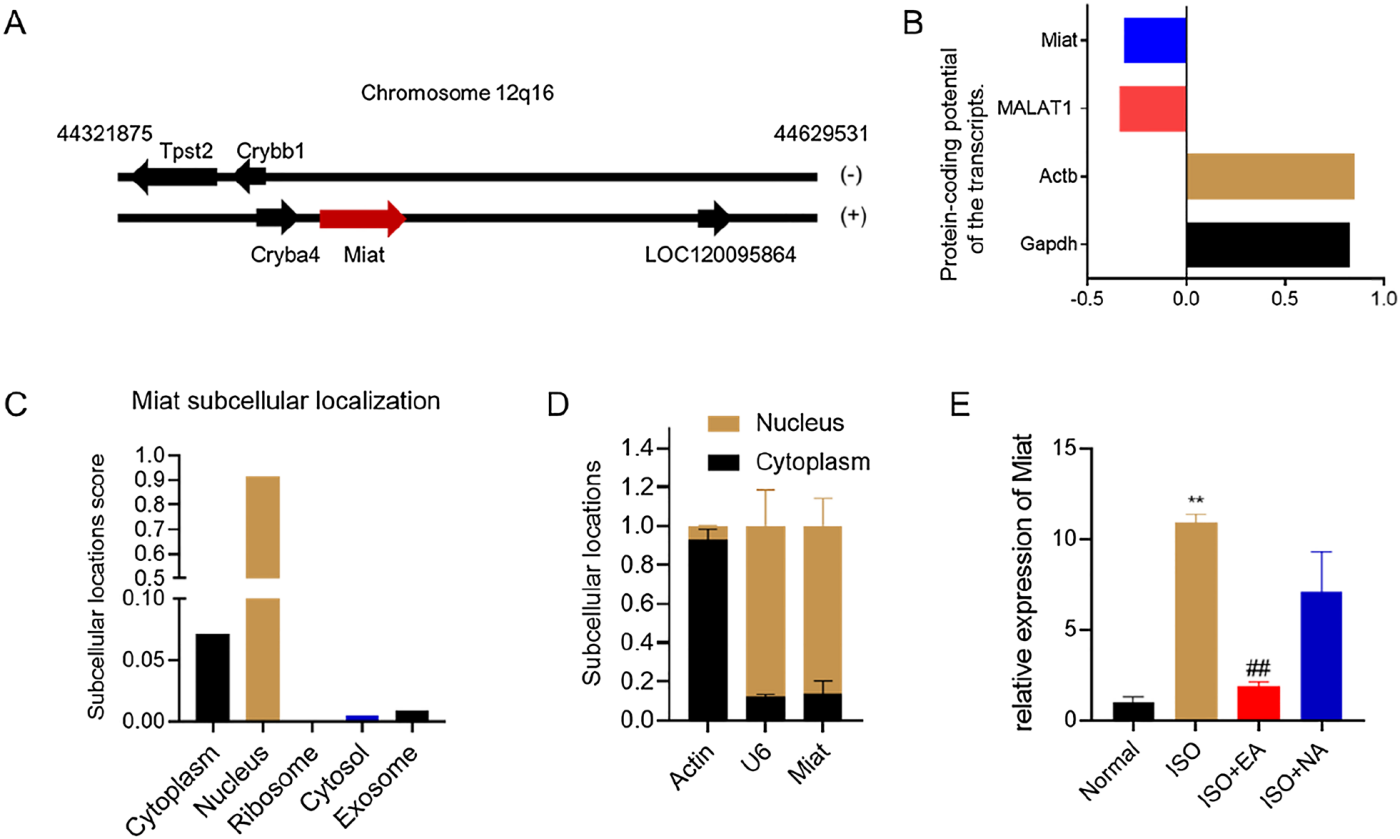
Electroacupuncture reduced the expression of lncRNA Miat. **A** Schematic illustration of the genomic locus of *Miat* and neighboring genes on rat chromosome 12q16. **B** *Miat* was predicted to be of a poor protein coding capacity. The RNA sequences of Miat, MALAT1, Actb and Gapdh were evaluated by the CNIT program. MALAT1 served as a control non-coding transcript, while Gapdh and Actb served as control protein coding transcripts. **C** Subcellular localization of Miat precited by the lncLocator program. **D** Nuclear separation experiments showed the subcellular localization of Miat. Actin mRNA was used as a control for the cytoplasmic fraction; U6 mRNA was used as a control for the nuclear fraction. **E** The relative expression levels of Miat were detected in different groups. Values are expressed as the means ± SD (n = 3), *P < 0.05, **P < 0.01

The rat *Miat* gene locus location is 12q16, and oriented sense configuration (Fig. 3A). Prediction of the Miat translation capacity using CNIT program [52] showed that the *Miat* protein-coding ability was poor (Fig. 3B). Using lncLocator program [53] to predict the Miat subcellular localization, the results showed that most of Miat is located in the nucleus (> 90%), and a small amount is located in the cytoplasm (> 7%) (Fig. 3C). Our nuclear separation experiments showed that the abundance of Miat was much higher in the nuclear fraction compared with the cytoplasmic fraction (Fig. 3D). We also examined the effects of electroacupuncture on Miat and found that electroacupuncture at PC6 decreased the expression level of Miat, which caused overexpression by ISO injection, while electroacupuncture at non-point had no significant change (Fig. 3E).

### LncRNA Miat sponges miR-133a-3p in an Ago2-dependent manner

**Fig. 4:**
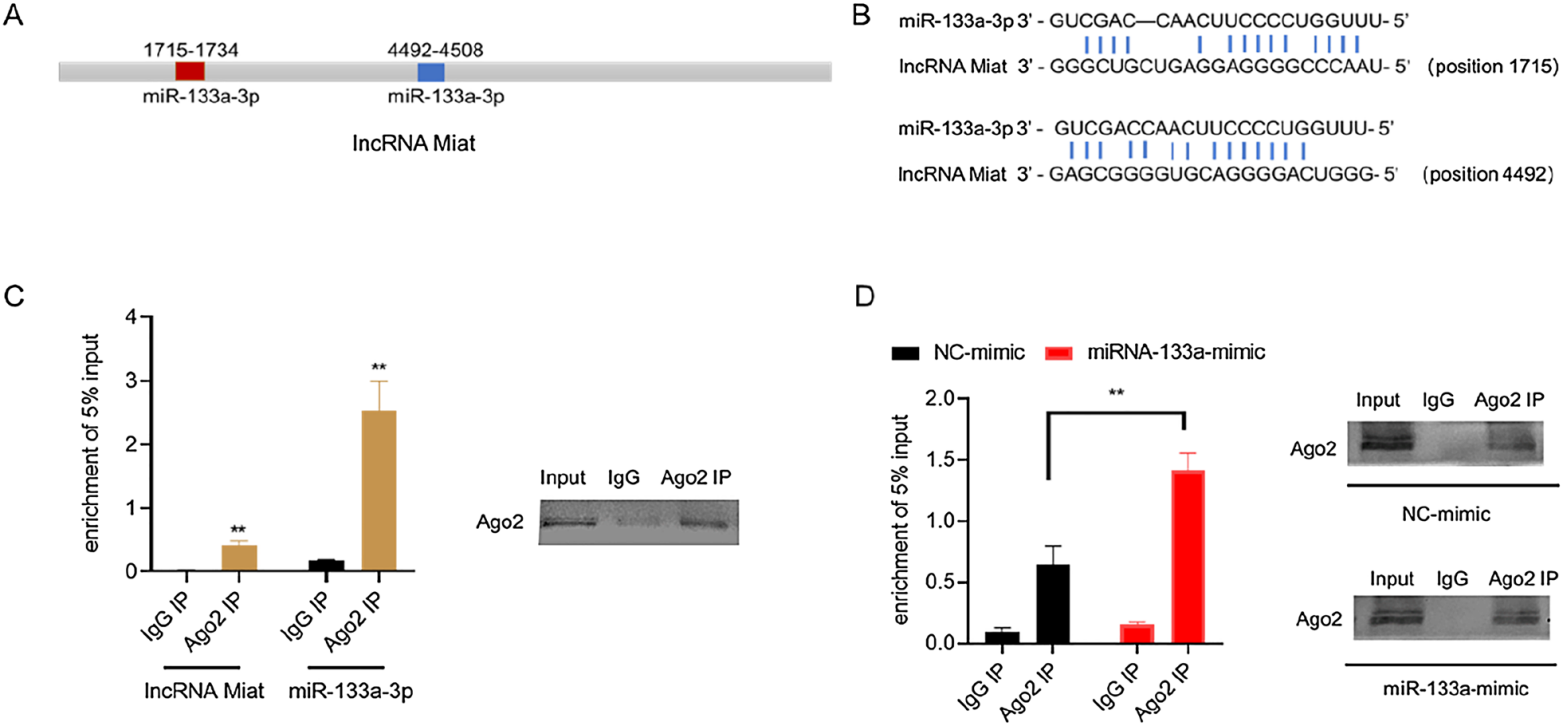
Miat sponges miR-133a in an Ago2-dependent manner. **A**, **B** Bioinformatics analysis of the possible binding sites between Miat and miR-133a. **C** The RIP assay and Western blot were performed to confirm whether Miat and miR-133a-3p could directly bind to Ago2 in H9c2 cells. **D** Anti-Ago2 RIP was performed in H9c2 cells transiently overexpressing miR-133a and followed by RT-qPCR and western blot to detect lncRNA-Miat associated with Ago2. Values are expressed as the means ± SD (n = 3), *P < 0.05, **P < 0.01

Bioinformatics predictions using the Targetscan website [54] indicated that the Miat sequence contained the putative binding site of miR-133a-3p (Fig. 4A and B). In addition, it has been previously reported that Miat is the direct target of miR-133a-3p [55], miRNA exists in the form of miRNA ribonucleoprotein complex (miRNP), which contains Ago2, the core component of RNA-induced silencing complex [56]. MicroRNAs bind to their targets and cause translational repression and/or RNA degradation in an Ago2-dependent manner [57]. To determine whether lncRNA Miat binds to miR-133a-3p in this manner, RNA-binding protein immunoprecipitation (RIP) was performed in H9c2 cardiomyocytes using Ago2 antibody (Fig. 4C). It was found that Miat and miR-133a-3p preferentially enriched miRNP containing Ago2 compared with IgG immunoprecipitates. In addition, after mimic-133a-3p was transfected with H9c2 cells, more Miat were employed by Ago2 (Fig. 4D). We further determined that Miat can bind to miR-133a-3p through an Ago2-based manner.

### Inhibiting the expression of CTGF through Miat-miR-133a-Ago2

**Fig. 5:**
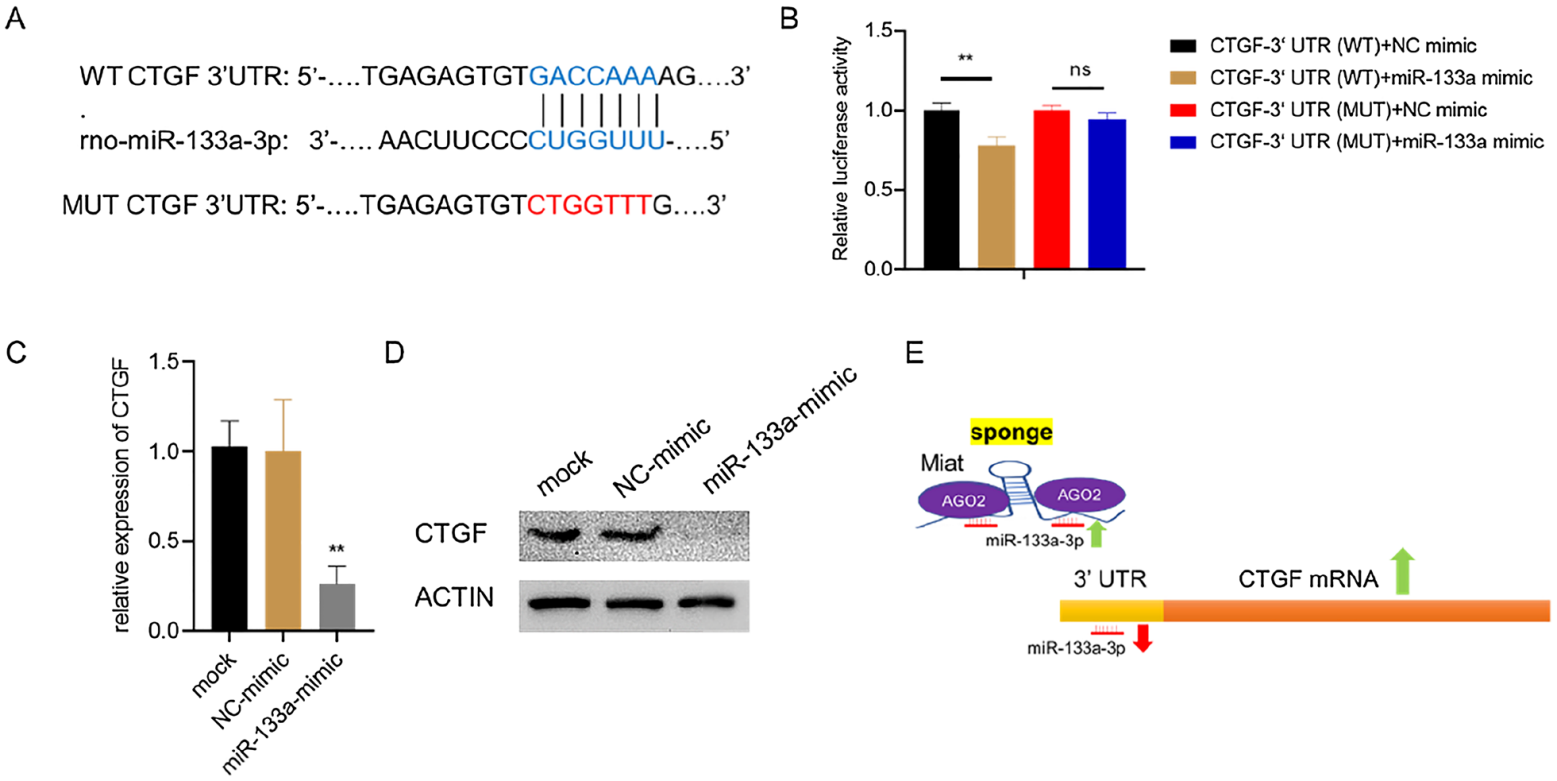
CTGF is a direct target of miR-133a-3p in H9c2 cells. **A** CTGF was predicted as a target gene of miR-133a-3p using Targetscan. **B** HEK293 cells were transfected with miR-133a-3p mimic and luciferase constructs of CTGF 3′-UTR (Wild Type, WT) or mutant CTGF 3′-UTR-MUT. Luciferase activity was detected 48 h after transfection. **C**, **D** H9c2 cells were transfected with miR-133a-3p mimic. The mRNA (**C**) and protein (**D**) expression levels of CTGF were determined by RT-qPCR and Western blot. **E** A schematic outline of how the lncRNA Miat may function as a ceRNA for miR-133a-3p-Ago2 and promote the expression of CTGF protein. Values are expressed as the means ± SD (n = 3), *P < 0.05, **P < 0.01

In order to further explore the myocardial fibrosis regulation effect of Miat on the sponge effect of miR-133a-3p, the Targetscan website was used to predict the binding of miR-133a-3p with wild-type CTGF-3ʹUTR (Fig. 5A). Subsequently, the relationship between CTGF and miR-133a-3p was confirmed by the luciferase reporter gene assay, and compared with the CTGF-3ʹUTR (WT) + NC mimic group, the relative luciferase activity of CTGF-3ʹUTR (WT) + miR-133a-3p mimic group was significantly decreased (Fig. 5B). In addition, the mutation of CTGF-3ʹUTR eliminated its binding to miR-133a-3p, meaning that miR-133a-3p binds to the wild-type CTGF-3ʹUTR at the predicted binding site. We identified CTGF expression in H9c2 cells transfected with mir133a-3p mimic or NC mimic. The results showed that the expression level of CTGF mRNA and protein level were significantly down-regulated in the miR-133a-3p mimic group (Fig. 5C and D), indicating that the up-regulation of miR-133a-3p inhibited the expression of CTGF. A schematic outline of how the lncRNA Miat may function as a ceRNA for miR-133a-3p-Ago2, promoting the expression of CTGF protein is shown in Fig. 5E. Therefore, it can be proved that miR-133a-3p directly targets CTGF mRNA.

### LncRNA Miat binding with peroxisome proliferator-activated receptor-gamma (PPARG)

**Fig. 6:**
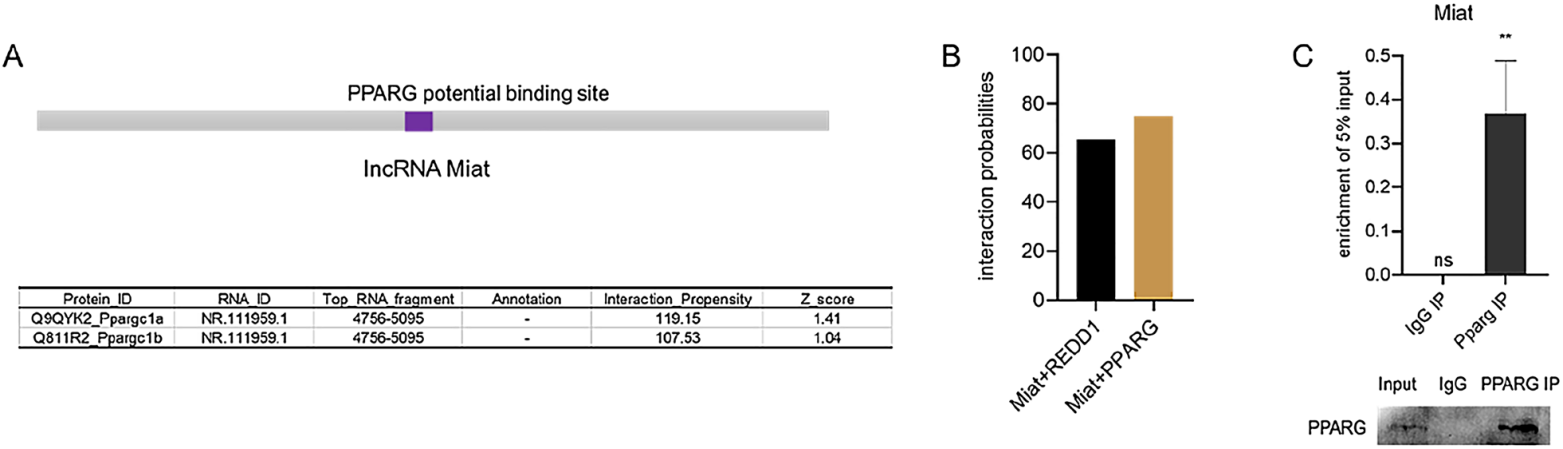
LncRNA Miat binding with the PPARG protein. **A** Using catRAPID program, an interaction between Miat and PPARG was predicted. **B** LncPro program was used to verify the combination between Miat and PPARG, while a validated combination of Miat and REDD1 was used as a control. **C** The combination between Miat and PPARG was detected by RNA immunoprecipitation and Western blot. Values are expressed as the means ± SD (n = 3), *P < 0.05, **P < 0.01

Most Miat exists in the nucleus, and the regulatory role of lncRNA Miat in the nucleus was explored. We used catRAPID [58] to predict the proteins interacting with Miat, and the results showed that PPARGc1a/1b, the coactivator of PPARG, was bound to lncRNA Miat. We further conducted a combination of PPARG and Miat using the lncPRO website [59], and the results showed that there might be a potential combination of Miat and the PPARG protein, and the predicted score was higher than the reported combination of Miat with the REDD1 protein [60] (Fig. 6B). Subsequently, RNA-IP experiments were further used to verify the combination of PPARG and Miat (Fig. 6C). Our results showed lncRNA Miat binding with the PPARG protein.

### Miat promotes the transcription of TGF-β1 by inhibiting the formation of heterodimer PPARG–RXRA complex

**Fig. 7:**
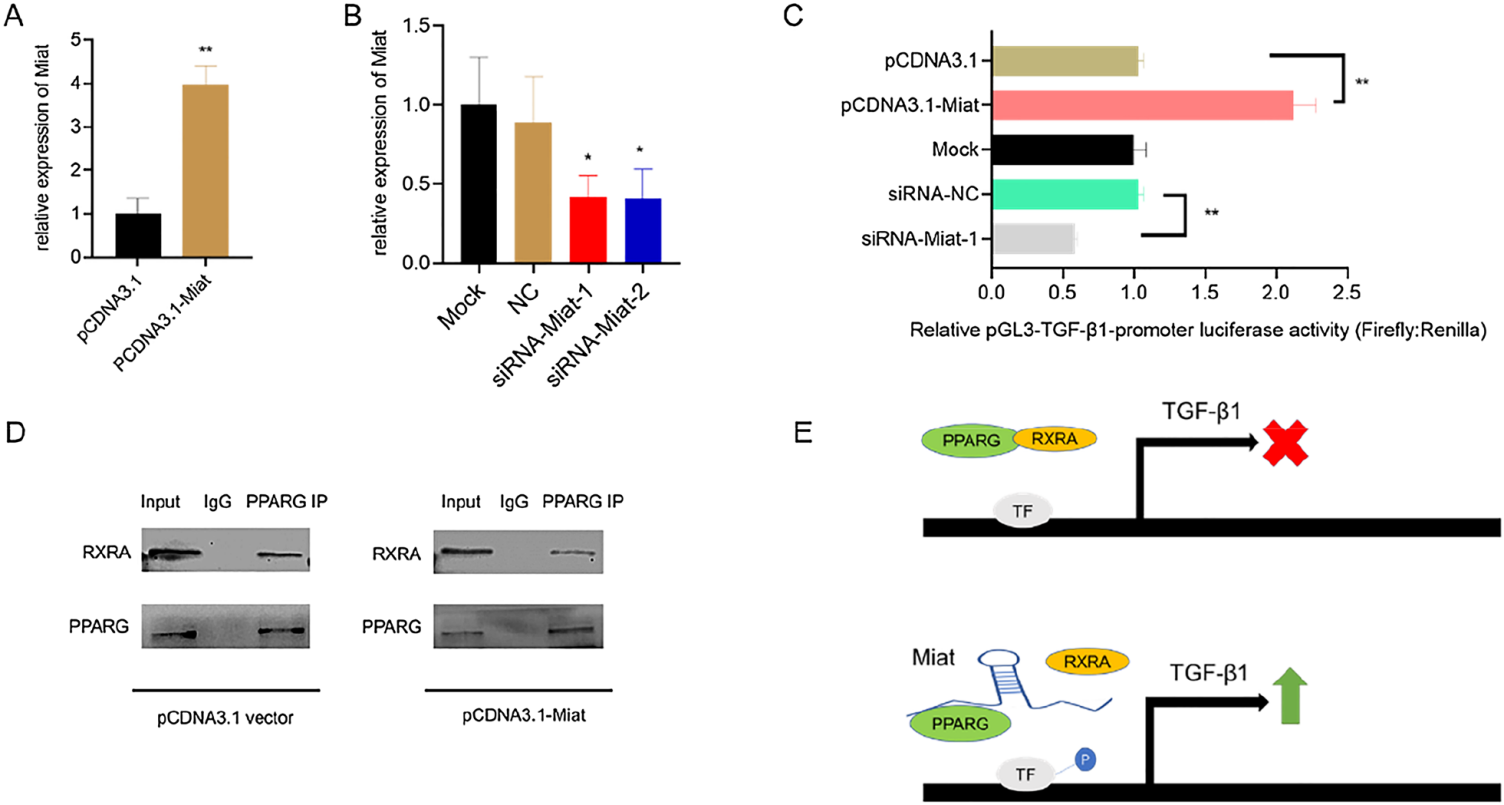
Miat inhibits the formation of the heterodimer PPARG–RXRA complex and promotes the transcription of TGF-β1. **A** Relative expression of Miat determined by RT-qPCR in H9c2 cells transfected with pcDNA3.1 or Miat overexpression vector. **B** RT-qPCR confirmed the down-regulation of Miat using two different siRNAs compared with the negative control (NC). **C** The dual-luciferase reporter assay detected the transcriptional activity of TGF-β1 with Miat overexpressing and knockdown. The values were normalized to control expression levels (n = 3). **D** Co-IP experiment was used to detect the binding between PPARG and RXRA with or without Miat overexpression. **E** Schematic illustration of the mechanism by which lncRNA-Miat acts as a decoy to weaken PPARG/RXRA interactions, inhibit TF (for example Zf9) dephosphorylation and promote the transcriptions of TGF-β1. Values are expressed as the means ± SD (n = 3), *P < 0.05, **P < 0.01

PPARG and RXRA are important heterodimers, and PPARG–RXRA has been shown to inhibit TGF-β1 gene through Zf9 dephosphorylation [61]. In order to determine whether Miat can participate in this regulatory process, we carried out knockdown and overexpression of Miat (Fig. 7A and B), and verified the activity of TGF-β1 promoter by dual-luciferase reporter gene assay. The results showed that knockdown Miat can reduce the activity of the TGF-β1 promoter, while overexpressing Miat can promote the activity of the TGF-β1 promoter (Fig. 7C). Then, in order to verify the correlation between Miat regulation of the TGF-β1 promoter activity and PPARG/RXRA, Miat was overexpressed in H9c2 cells, and co-IP assays results showed that the binding of PPARG/RXRA complex was weakened (Fig. 7D), PPARG/RXRA complex is involved in phosphorylation modification of transcription factors, for example Zf9 [61], thereby affecting the transcription capacity of TGF-β1. Based on the above results, we inferred that Miat was involved in regulating the TGF-β1 transcriptional efficiency by affecting the formation of heterodimer of PPARG/RXRA (Fig. 7E).

## Discussion

**Fig. 8:**
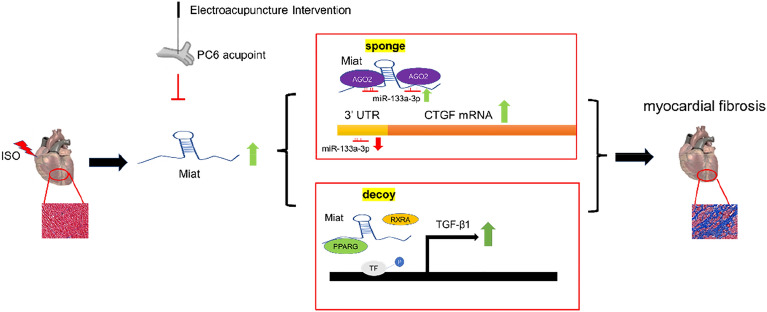
Schematic illustration of the mechanism by which the down-regulation of lncRNA Miat by electroacupuncture protects against myocardial fibrosis. The injection of ISO can lead to the occurrence of myocardial fibrosis and increase the expression level of Miat, which can adsorb miR-133a-3p as a sponge and increase the expression level of the CTGF protein. Miat can also be used as decoy to promote the separation of the heterodimer PPARG/RXRA and improve the transcription of TGF-β1, thus causing myocardial fibrosis. Electroacupuncture PC6 can inhibit the expression of Miat and inhibit myocardial fibrosis

Due to the difference in its expression in the heart under normal and pathological conditions, Miat has recently attracted great interest among researchers [62–64]. Our results showed that electroacupuncture can inhibit myocardial fibrosis by reducing the expression of Miat (Fig. 8). LncRNA Miat plays a dual regulatory role during myocardial fibrosis, it acts as “sponge” and “decoy”. At the post-transcriptional level, Miat sponges miR-133a-3p and inhibits its targeting of the CTGF mRNA; the sponge effect between Miat and miR-133a-3p is based on an Ago2-dependent manner. Additionally, Miat can decoy the PPARG protein and promote the dissociation of the heterodimer PPARG and RXRA complex in the nucleus. Miat attenuates the inhibitory effect of PPARG and RXRA complexes on TGF-β1 promoter, thus promoting the TGF-β1 transcription. Above all, Miat is a very effective target to reduce myocardial fibrosis. Reducing the expression of Miat by electroacupuncture may be an effective way to inhibit myocardial fibrosis.

It has been reported that acupuncture can effectively inhibit the production of ET-1 and the mRNA expression of type I and III collagen [65]. Electroacupuncture at Neiguan acupoint can promote the stem cell survival and improve ischemic heart function. Electroacupuncture could become a useful approach in stem cell therapy for ischemic heart diseases [66]. In a male chimpanzee (Pan Troglodytes) diagnosed with frequent premature ventricular contracts, acupuncture and laser therapy (PC6 and HT acupoints) appeared to decrease the mean number of VPC/min in this larger state. The rat model of chronic myocardial ischemia was established by subcutaneous injection of isoproterenol (ISO) for 14 days. Electroacupuncture pretreatment promoted angiogenesis by increasing serum HIF-1α and VEGF protein expression in myocardial infarction area [67]. ISO is often used to construct models of heart disease in rats, e.g., heart failure (HF) [68], cardiac hypertrophy [69] and chronic myocardial ischemia [67]. At the same time, ISO injection and left anterior descending (LAD) ligation are commonly used to construct rat models of myocardial fibrosis [70–72]. Although surgical modeling such as ligation of the LAD coronary artery has been widely used, the period is short and fast, but with high mortality. Animal models of drug-induced myocardial fibrosis have good repeatability, low mortality and are relatively stable. Therefore, in this study, we use ISO to build the rat model of myocardial fibrosis.

Compared with many other easily mutated lncRNA, Miat is highly evolutionarily conserved and highly expressed in mouse brains and continuously expressed in adult human brain [30, 64]. LncRNA Miat is susceptible to a variety of harmful factors, such as angiotensin II, isoproterenol, hypoxia and infection; Miat is anomaly overexpressed in serum, plasma, blood cells and myocardial tissues under a variety of cardiovascular conditions including myocardial infarction, cardiac hypertrophy and atrial fibrillation [36, 63, 73]. Studies have shown that lncRNA can perform biological functions regardless of whether they are in the nucleus or cytoplasm [74, 75]. In the cell nucleus, lncRNA mainly regulates chromatin, transcription regulation and variable shear regulation. In the cytoplasm, the ceRNA regulation mechanism that adsorbs miRNA is mainly used to affect mRNA stability and translation regulation [76]. Miat usually acts as an endogenous spongiform that competitively binds to miRNA and thus plays an inhibitory role on mRNA [77]. For example, knockdown Miat could up-regulate the miRNA-24 expression and reduce the expression of TGF-β1 [78]. There are three binding sites of miRNA-150 in Miat, and Miat can regulate the expression of P300 through competitive binding with miRNA-150, thus regulating the occurrence of isoprenaline-induced cardiac hypertrophy [79]. Previous reports have found that Miat can sponge miRNA-133a-3p [55]. Our study further showed that Ago2 protein could simultaneously bind to Miat and miRNA-133a-3p by RNA-IP assay. In addition, after the overexpression of miRNA-133a-3p, Ago2 protein-bound Miat significantly increased, thus proving that lncRNA Miat sponges miRNA-133a-3p through an Ago2-dependent manner. In the nucleus, lncRNA can act as decoys and lure transcription factors away from the specific location. Through nuclear and cytoplasmic separation and qPCR experiments, we found that Miat is actually rich in nuclei. This also implies that Miat plays a more important regulatory role in the nucleus. PPARG is a transcription factor known to have antidiabetogenic and immune effects, and PPARG heterodimerizes with the retinoid X receptor (RXR). This complex recognizes PPAR response elements (PPRE) in promoters on target genes resulting in the regulation of gene transcription [80, 81].

It is predicted that Miat may bind to PPARG, and PPARG/RXRA heterodimer regulates TGF-β1 transcriptional activity. The research results showed that PPARG activation might trans-repress the *TGF-β1* gene, thereby altering the expression of TGF-β-inducible target genes [61]. The activation of the PPARG and RXR heterodimer contributes to the gene regulation [82]. We used dual-luciferase assay to confirm that Miat was involved in regulating the transcriptional activity of TGF- β1 promoter. This regulation may be based on the fact that Miat acts as a decoy to dissociate the binding of PPARG and RXRA heterodimer and may influence the heterodimer function on the transcriptional activity of TGF-1. In the promoter region of the TGF-β1 gene, the putative binding sites for PPARG–RXRA seemed to be unactive. The TGF-β 1 gene contains DNA reaction elements that interact with Zf9 [83, 84]. The activation of PPARG–RXR has been shown to inhibit the *TGF-β1* gene through Zf9 dephosphorylation [61]. We hypothesized that the overexpression of Miat reduces the binding efficiency of PPARG and RXR, thereby affecting the phosphorylated form of Zf9. Ultimately, the transcriptional efficiency of TGF-β1 is affected. LncRNA has a variety of regulatory effects, but few reports revealed that lncRNA can affect the occurrence and progression of diseases through the combination of multiple regulatory effects. Therefore, based on the above results, Miat can act as a sponge to adsorb miRNA and regulate the expression of CTGF mRNA and act as a protein decoy to regulate the transcriptional activity of TGF-β1 at chromatin level.

## Conclusion

We revealed that electroacupuncture at PC6 point can inhibit the process of myocardial fibrosis by reducing the expression of lncRNA Miat. Miat can not only act as a molecular sponge to regulate the binding of miRNA-133a to CTGF mRNA, but can also act as a decoy affecting the formation of the heterodimer PPARG and RXRA protein complex. This dual regulatory effect shows the important functions of lncRNA. In future studies, more lncRNA with multiple regulatory functions will be investigated and studied. At the same time, this work provides more insights for an in-depth study of the molecular mechanism of electroacupuncture in the treatment of cardiovascular diseases.

## Supplementary Information

**Additional file 1: Figure S1. **Uncropped image of blots and gels in the article. **Table S1.** RT-qPCR primers and siRNA oligonucleotides used in this study.

## Data Availability

The datasets used and/or analyzed during the current study are available from the corresponding author upon reasonable request.

## Abbreviations

Miat: Myocardial infarction associated transcript
PC6: Neiguan point
lncRNA: Long non-coding RNA
ISO: Isoproterenol treated
RT-qPCR: Real time-quantitative reverse transcription PCR
PPARG: Peroxisome proliferative activated receptor, gamma
RXRA: Retinoid X receptor alpha
UTR: Untranslated region
CTGF: Connective tissue growth factor
TGF-β1: Transforming Growth Factor Beta 1
NF-κB: Nuclear factor kappa-light-chain-enhancer of activated B cells
TNF-α: Tumor necrosis factor-α
MMP-9: Matrix metalloprotein9
EA: Electroacupuncture at PC6
NA: Electroacupuncture at non-acupoint
WT: Wildtype
MUT: Mutation
RIP: RNA immunoprecipitation
Co-IP: Co-immunoprecipitation
ECG: Electrocardiogram
miRNP: MiRNA ribonucleoprotein complex
Ago2: Argonaute RISC Catalytic Component 2
HIF-1α: Hypoxia duciblefactors-1 alpha
VEGF: Vascular endothelial growth factor
LAD: Left anterior descending
DMEM: Dulbecco’s modified Eagle’s medium
HE: Hematoxylin–eosin
HRP: Horseradish peroxidase
IHC: Immunohistochemistry
NC: Negative control
PBS: Phosphate buffered saline
SD: Standard deviation
