# Supplementary material for: Downregulation of lncRNA Miat contributes to the protective effect of electroacupuncture against myocardial fibrosis
Source: Chin Med. 2022 May 17;17:57. doi: 10.1186/s13020-022-00615-6 (PMC9112552; doi:10.1186/s13020-022-00615-6)
Supplement: Supplementary file 1 — Additional file 1: Figure S1. Uncropped image of blots and gels in the article. Table S1. RT-qPCR primers and siRNA oligonucleotides used in this study. [file 13020_2022_615_MOESM1_ESM.docx]

Figure S1. Uncropped image of blots and gels in the article


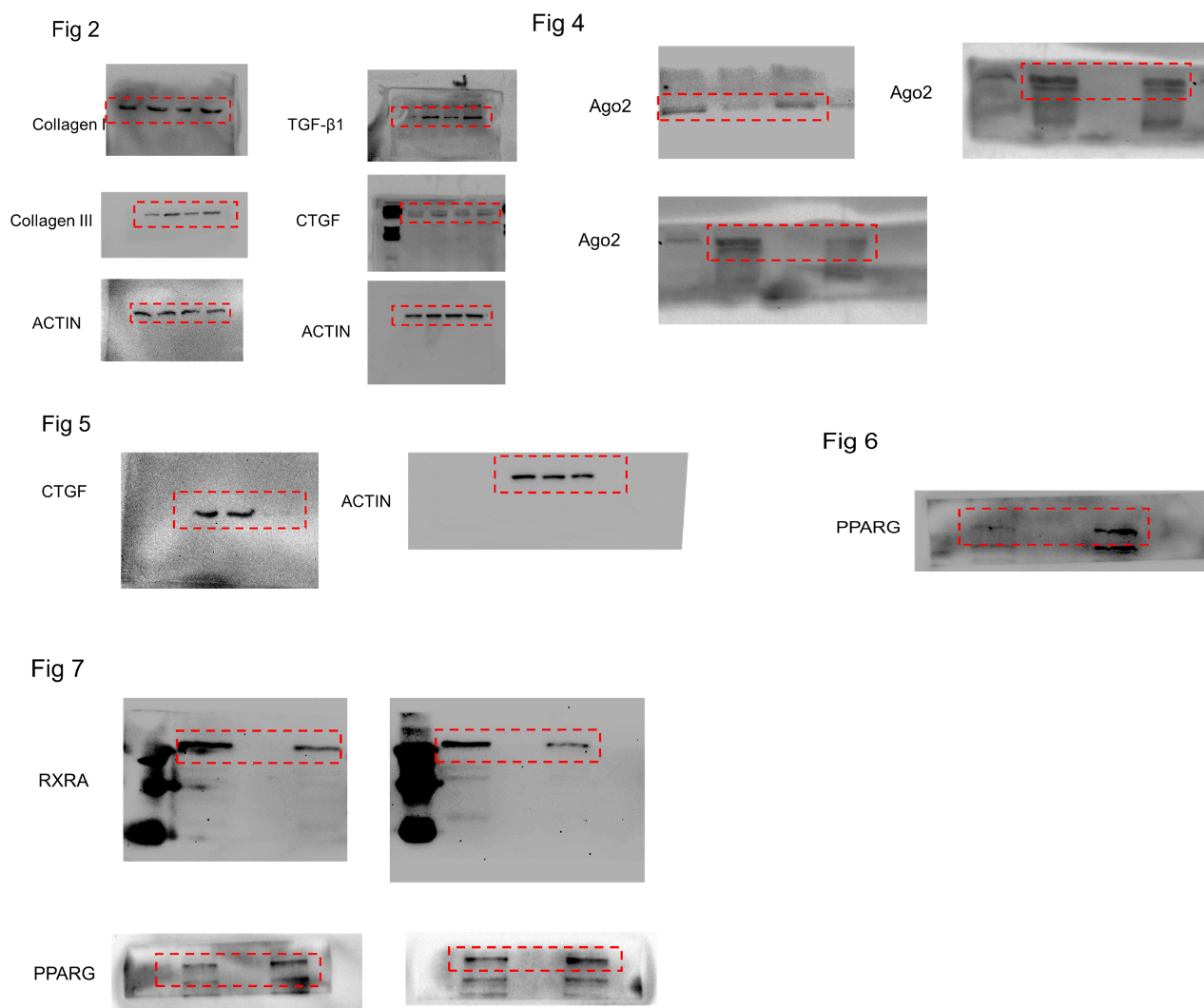


Table S1. RT-qPCR primers and siRNA oligonucleotides used in this study

| Name Sequence（5'-3'） | |
| --- | --- |
| MIAT Rat F | TTTCTGCCTGTTTGCTGCTT |
| MIAT Rat R | CCTCCCTACTGGGTCTCCTT |
| TGF-β1 Rat F | AACAATTCCTGGCGTTACCT |
| TGF-β1 Rat R | GCCCTGTATTCCGTCTCCTT |
| Collagen I Rat F | TCCTGCCGATGTCGCTATCC |
| Collagen I Rat R | TCGTGCAGCCATCCACAA |
| Collagen III Rat F | GCCTTCTACACCTGCTCCTG |
| Collagen III Rat R | AGCCACCCATTCCTCCGACT |
| CTGF F | AGAGTGGAGATGCCAGGAGA |
| CTGF R | CACACACCCAGCTCTTGCTA |
| Rat Actin F | cacccgcgagtacaaccttc |
| Rat Actin R | cccatacccaccatcacacc |
| siMiat-1 | GGUGUGAUUAACCUACUAATT |
| siMiat-1 | UUAGUAGGUUAAUCACACCTT |
| siMiat-2 | GCAAGUGGUUCUUAGCUCATT |
| siMiat-2 | UGAGCUAAGAACCACUUGCTT |
